# Phyto-synthesis and structural characterization of catalytically active gold nanoparticles biosynthesized using *Delonix regia* leaf extract

**DOI:** 10.1007/s13205-016-0432-8

**Published:** 2016-06-02

**Authors:** Preeti Dauthal, Mausumi Mukhopadhyay

**Affiliations:** Department of Chemical Engineering, S.V. National Institute of Technology, Surat, 395007 Gujarat India

**Keywords:** *Delonix regia*, Biofabrication, Gold, Nanoparticle, Nitroaniline

## Abstract

Biological methods of nanoparticles synthesis are ecologically sound and sustainable alternative to the conventional methods. On the basis of aforesaid premise, the present study deals with the optimization and fabrication of gold nanoparticles (Au-NPs) using easily available bio-resource, *Delonix regia* leaf extract. The use of practically nontoxic natural extracts and water allows the synthesis pathways presented to be considered as ‘‘green’’ and so permitting the synthesized Au-NPs to be used in sensitive areas, such as bioremediation. Various characterization techniques are adopted for the evaluation of size, stability, morphology, crystal nature, and purity of nanoparticles. Ultraviolet–visible spectroscopy analysis showed a surface Plasmon resonance peak for prepared Au-NPs at 542 nm, and its absorbance increased with increasing the interaction time. Transmission electron microscopy analysis showed that the particles were spherical and 4–24 nm in size. Energy dispersive X-ray spectroscopy analysis displayed a 2.2 keV peak corresponding to the pure phase gold nanocrystal. X-ray diffraction analysis proved the fabrication of crystalline Au-NPs with face-centered cubic geometry within 10 min. Furthermore, ζ potential (−15 mV) and Fourier transform infrared data suggested the role of polar polyphenolic compounds of leaf extract in fabrication and stabilization process. Biofabricated nanoparticles are demonstrated to have catalytic activity for the reduction of toxic nitro-organic pollutant *o*-nitroaniline. Therefore, the present study offers a straightforward, cost-efficient, eco-friendly, and sustainable alternative for the fabrication of catalytically active Au-NPs.

## Introduction

Nanotechnology has significantly impacted every facet of human life ranging from pharmaceutical to cosmetics, clinical diagnostics to therapy, and pollution detection and their remediation. Every aspect of human life has been revolutionized with the advancements of nanotechnology in the area of metallic nanoparticles fabrication and their applications. Metallic nanoparticles exhibit a complete range of new and improved size- and shape-dependent physicochemical and biological properties that are significantly different from bulk counterparts (Singh et al. 2015). The material particles generally show fascinating and even unanticipated properties when they are in the nanorange of 1–100 nm. Noble metals, such as of gold, palladium, platinum, and silver with nano-size dimensions, have received considerable attention due to their endless promises in the field of material science (Dauthal and Mukhopadhyay 2013a). Among these noble metal nanoparticles (NPs), particularly gold nanoparticles (Au-NPs) play a significant role in various catalytic reactions due to high-surface area-to-volume ratio, energetic surface atoms, and great surface energy (Bai et al. 2009). Traditionally, gold nano-catalysts are fabricated through various physical and chemical methods. Although existing methods have successfully produced well-defined NPs, the concerns regarding environmental pollutions and requirement of high-cost instrumental setups also increase simultaneously. The astringent reaction conditions due to the use of highly toxic and inflammable chemicals make these conventional processes unsuitable for various biological applications and restrict their scaling-up for industrial applications.

Biofabrication methods are not only a noble approach to synthesize benevolent nano structures, but also a footstep to diminish the utilization of toxic chemical and generation of perilous by-products which are hazardous to environment and public health (Jacob et al. 2012). These methods are considered as benign, sustainable, cost effective, and ecologically sound. Biofabrication of Au-NPs using microorganisms, such as bacteria (Alani et al. 2012; Srivastava and Mukhopadhyay 2014; Wang et al. 2015), fungi (Metuku et al. 2014), virus (Slocik et al. 2005), yeast (Agnihotri et al. 2009), and algae (Rajathi et al. 2012), are already reported. However, the use of the plant extract as the potential resource for the fabrication of NPs is gaining a significant importance, as unlike microbial fabrication, these methods do not involve isolation and culture preparation techniques (Gardea-Torresdey et al. 2003; Tripathy et al. 2010).

In a recent study, antioxidant potential of *Delonix regia* (*D. regia*) has been utilized for cost effective and eco-benign fabrication of palladium and gold–palladium NPs (Dauthal and Mukhopadhyay 2013b, 2016). Metal-reducing potential of one other plant species of *Delonix* genus is also reported recently for the synthesis of silver NPs (Sathiya and Akilandeswari 2014). However, literature survey revealed that there is no report available for the biofabrication of Au-NPs using *D. regia.* Thus, the present study is intended to fabricate Au-NPs utilizing the metal-reducing potential of *D. regia* extract. To further strengthen this biofabrication approach, the effects of various process parameters, such as leaf extract concentration, temperature, time, and pH of the reaction, are also investigated for the fabrication of NPs. Furthermore, the catalytic potential of biofabricated NPs was evaluated for the reduction of anthropogenic pollutant *o*-nitroaniline (*o*-NA). The use of biofabricated NPs in this direction could be seen as a prominent alternative for traditionally synthesized polymer supported nanocatalyst (Zayed and Eisa 2014; Zayed et al. 2015), where the structure and functional group of polymer support influences the catalytic behavior of NPs (Liu et al. 2007). However, in the case of biofabricated NPs, surface biomolecules (functional groups) can act as a polymer support, and influence of external polymer support on catalytic activity of NPs can be minimized (Sharma et al. 2007; Gangula et al. 2011; Dauthal and Mukhopadhyay 2012, 2013b, 2015a, b). Furthermore, the inert behavior of supported biomass with an added advantage of eco-friendliness makes biofabricated NPs as noble tool for bioremediation.

## Experiments

### Materials

Tetrachloroauric (III) acid (HAuCl_4_·3H_2_O, 99 %), sodium borohydride (NaBH_4_, 98 %), and *o*-Nitroaniline (C_6_H_6_N_2_O_2_, 98 %) were procured from HiMedia Pvt. Ltd. Mumbai, India. Fresh leaves of *D. regia* were collected from the campus of S.V. National Institute of Technology Surat, Gujarat, India.

### Biofabrication of Au-NPs

30 g of fresh leaf of *D. regia* was mixed with 120 mL of deionized water and heated at 60 °C for 10 min and then filtered. This solution was decanted and stored at 4 °C for further use. About 100 mL of aqueous *D. regia* leaf extract was added to 800 mL of 1 × 10^−3^ M HAuCl_4_·3H_2_O solution. This solution was kept at room temperature in a sealed flask, for 10 min. For control experiments, the same amount of *D. regia* leaf extract and HAuCl_4_·3H_2_O solution was maintained separately at the same reaction environment. The reduced Au-NPs were sonicated for 10 min to separate Au-NPs from the bio-organics of *D. regia* leaf extract. Repeated centrifugation (14,000 rpm for 10 min) was performed after sonication. After centrifugation, pellets were washed with deionized water and dried followed by characterization using different techniques.

### Catalytic reduction of *o*-NA

In a glass vial, 3 mL of *o*-NA (0.5 × 10^−3^ M) was mixed with 0.12 mL of NaBH_4_ (1.0 M) and 1 × 10^−3^ g of nanocatalyst (Au-NPs) at 28 ± 2 °C. The catalytic reduction of *o*-NA was monitored using UV–visible spectrophotometer. Catalytic reduction of *o*-NA was carried out using different concentration levels of Au-NPs (0.5–2 × 10^−3^ g) and reaction temperature range (10–50 °C). To better understand the catalytic activity of NPs, blank reaction was also carried out for the reduction of *o*-NA in the absence of Au-NPs. To judge the recyclability of catalyst, the Au-NPs were separated from the reaction mixture by centrifugation followed by washing and drying and then reused for five cycles using the above-mentioned procedure.

### Characterization

Optical absorbance of biofabricated Au-NPs was monitored in DR 5000, HACH, USA UV–visible spectrophotometer. ζ potential and hydrodynamic size distribution of NPs were determined using Zetasizer Nano ZS90, Malvern, UK instrument. CM200, Philips, UK; TEM was used for TEM imaging and selected area electron diffraction pattern (SAED) measurements. X’Pert Pro, PANalytical, Holland was used for the analysis of XRD spectrum of NPs. Elemental analysis was carried out using EDX detector (INCAX-sight, Oxford, UK) coupled with SEM (JSM-6380LV, JEOL, Japan). FTIR analysis of Au-NPs was performed on FTIR, MAGNA 550, Nicolet, USA.

## Results and discussion

### UV–visible analysis

**Fig. 1:**
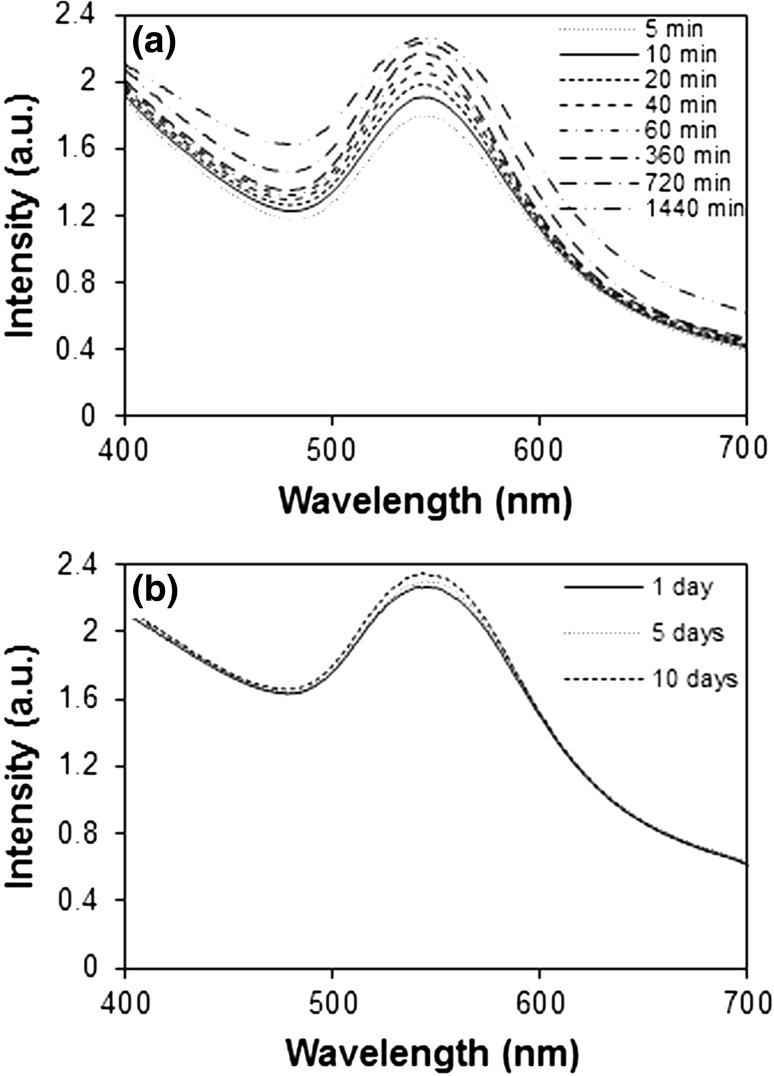
UV–visible spectra of *D. regia*-mediated colloidal Au-NPs (**a**) interaction time 5–140 min (**b**) long-term interaction study (1–10 days)

UV–visible spectra of biofabricated Au-NPs as a function of interaction time (5–1440 min) are represented in Fig. 1a. It was observed that the gold SPR peak observed at 542 nm. Intensity of this SPR peak was gradually increased as a function of interaction time without any significant change in the SPR position. Fabrication of Au-NPs occurs fairly rapidly. No significant change detected in position and intensity of SPR even after 720 min of incubation. The appropriate maximum absorbance was found to be 2.234–2.259 for 720–1440 min. This confirmed the complete reduction of Au^3+^ to Au-NPs within 720 min (12 h). Since more than 85 % conversion of Au^3+^ to Au-NPs was completed within first 10 min of the reaction time; therefore, 10 min of reaction time was considered for the synthesis of Au-NPs.

The long-term stability and aging effect on biofabricated Au-NPs was evaluated by monitoring its SPR position and ζ potential over a time period of 10 days. No significant change was observed in the SPR position and intensity (Fig. 1b), suggested the long-term stability of NPs.

The ζ potential was found to be −14.3 mV even after 10 days of incubation period, gave an additional proof of the long-term stability of Au-NPs. A negative value of ζ potential indicated the existence of repulsion force among particles and thereby increasing the stability. Thus, it may be concluded that there was not much agglomeration of the Au-NPs even after preserving solution for 10 days, validating the stability of NPs.

### DLS analysis

**Fig. 2:**
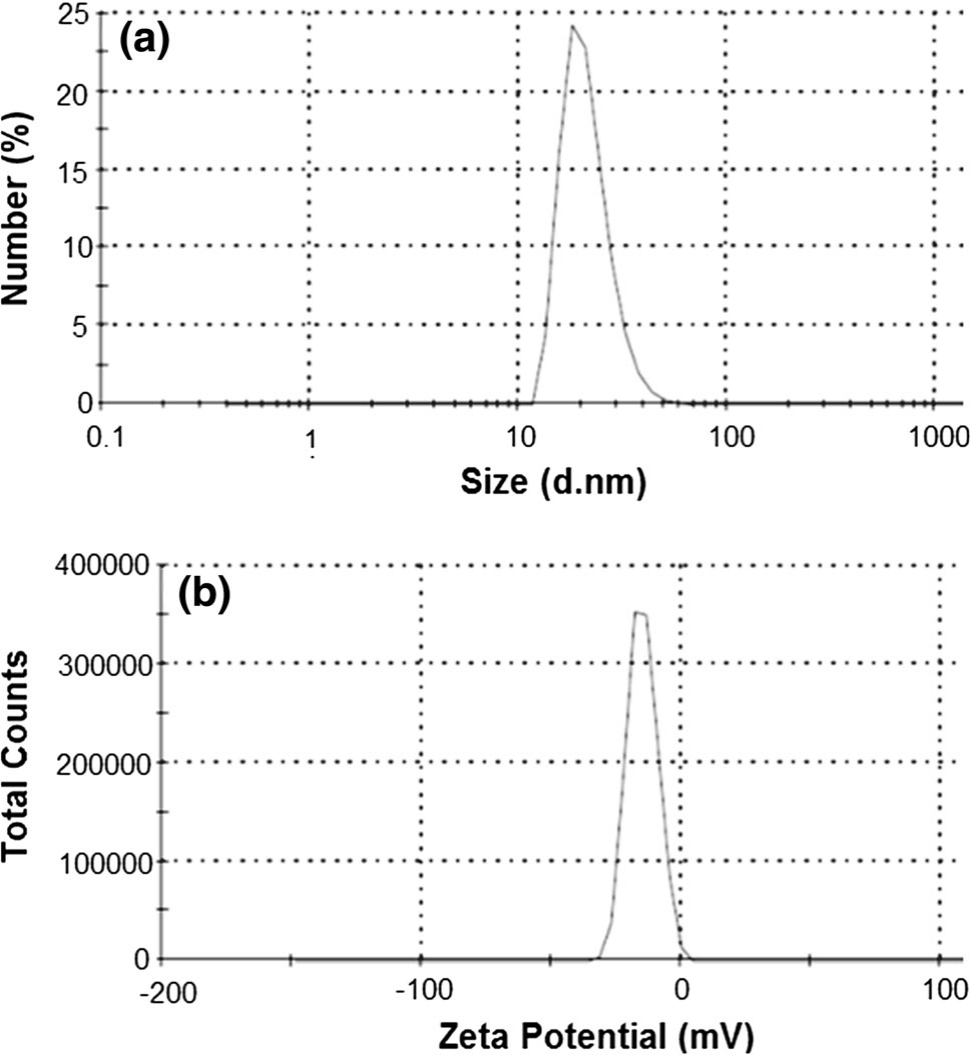
Biofabricated Au-NPs (**a**) hydrodynamic size (**b**) ζ potential distribution

DLS data represented the distribution of NPs in the range of 12–55 nm (average size 39 nm) with polydispersity index 0.279, which appears to be higher as compared to TEM (4–24 nm) and XRD measurements (25.77 nm) (Fig. 2a). This was due to interference created by electrical double layer on charged particles and overlapping NPs. However, the aggregation of NPs was avoided in the TEM imaging technique lead more precise observation (Suresh et al. 2011). In overall, the results of ζ potential value for Au-NPs obtained from colloidal solution were −15 mV, suggested the stability of biofabricated NPs (Fig. 2b).

### TEM analysis

**Fig. 3:**
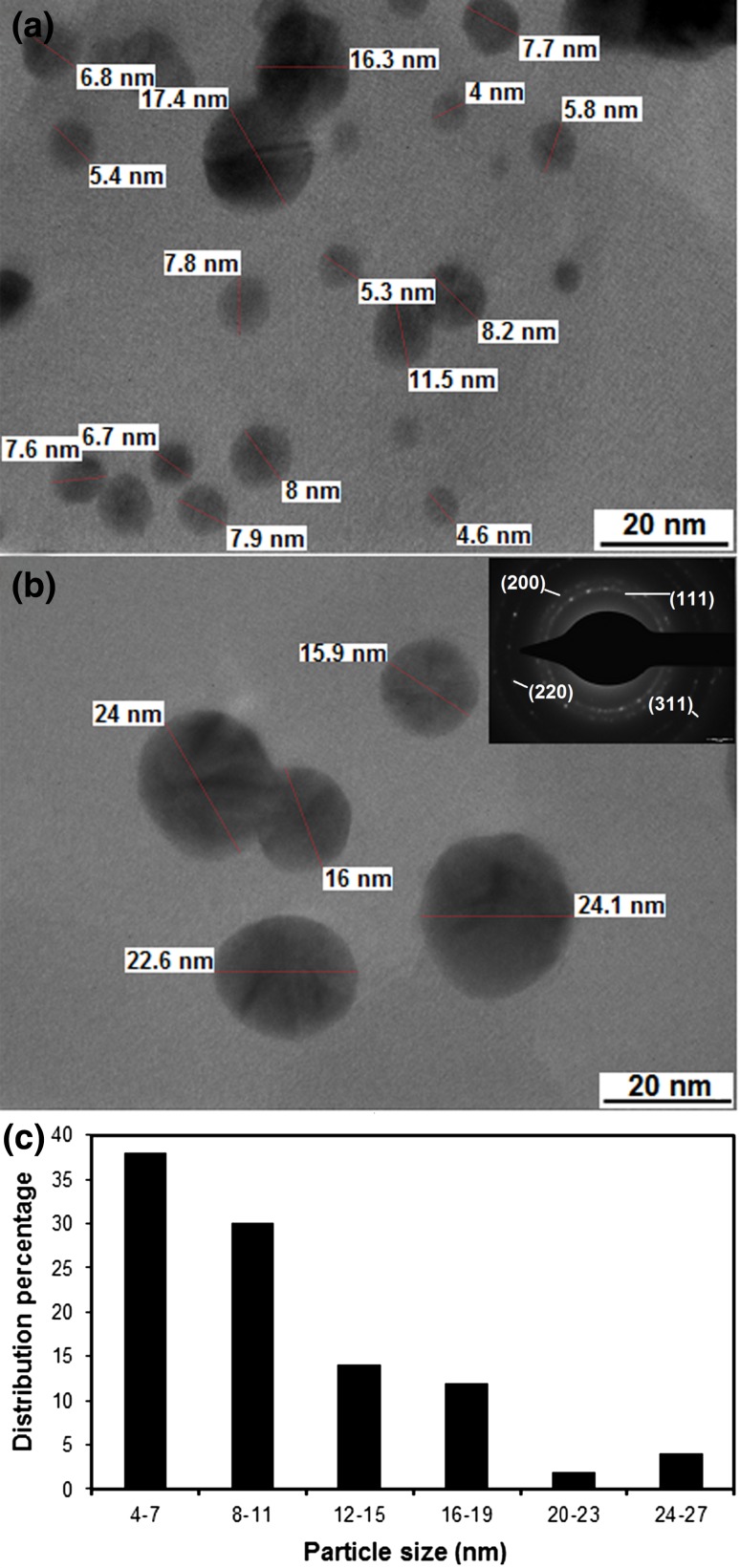
Surface morphology and size analysis of Au-NPs (**a**–**b**) TEM (*scale bar* 20 nm) (**c**) size distribution histogram (SAED pattern at inset)

TEM micrograph (Fig. 3a, b) clearly represented a well-dispersed assembly of Au-NPs ranged in dimension between 4 and 24 nm. Figure 3c showed the size distribution histogram of the nanoparticles. This histogram was prepared by manual analysis of 100 nanoparticles. Histogram indicated the average size (*µ*) of the prepared Au-NPs with the standard deviation (*σ*) (10.74 ± 5.23 nm). Histogram obtained from TEM micrograph of Au-NPs suggested that the majority of the particles (68 %) were resided in the size range of 5.51 nm (−1σ) and 15.97 nm (+1σ). Au-NPs predominately adopted a spherical morphology. No other shapes were observed, suggested the role of oxidized polyphenols of *D. regia* as stabilizing and capping agent for freshly fabricated Au-NPs, which avoided further growth. This observation was also supported by the FTIR and ζ potential analysis. It was also visible that the edges of the NPs were lighter than center, suggested capping and adherence of bio-organics of leaf extract on the surface of NPs (Ahmad et al. 2010). The crystalline structure of the Au-NPs was also detected by SAED pattern (inset in Fig. 3b). Well-defined spotty rings in SAED pattern represented the (111), (200), (220) and (311) reflections of crystalline Au-NPs.

### XRD analysis

**Fig. 4:**
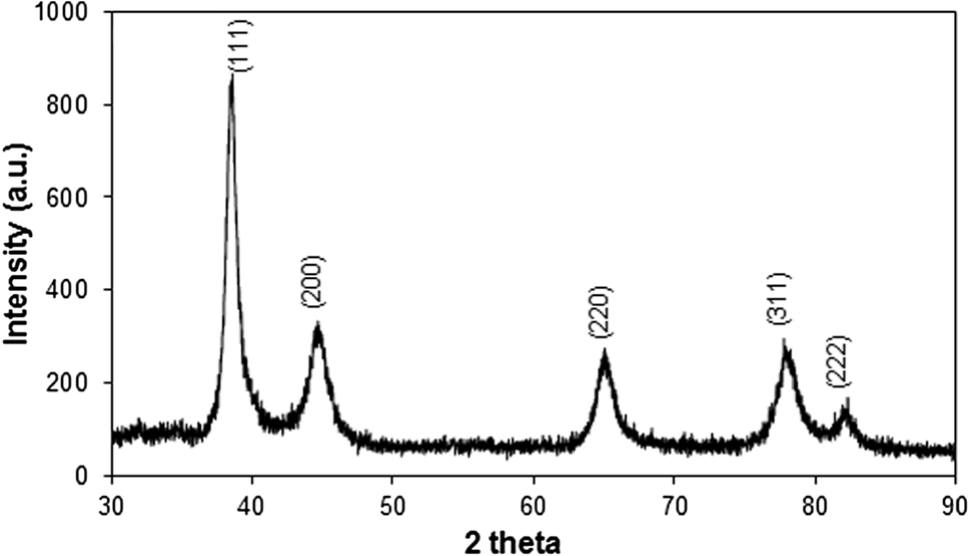
Principal Bragg reflections of biofabricated Au-NPs

The phase of the biofabricated Au-NPs was investigated by the XRD analysis and corresponding XRD pattern represented in Fig. 4. Figure represented clear Bragg’s reflections of face-centered cubic (fcc) phase of Au-NPs present at 38.46° (111), 44.43° (200), 65.01° (220), 77.81° (311), and 82.25° (222) positions (JCPDS No. 04–0784). The Bragg peaks of the Au-NPs are considerably broadened due to the small size of the nano-crystallites. The relative intensity of the (200)–(111) Bragg’s reflections was higher than the conventional value (0.45) suggested (111) face-enriched Au-NPs. Lattice constant calculated from this pattern was 4.069 Å established fcc geometry of NPs. Average particles size was calculated to be 25.77 nm using the line width of dominating (111) Bragg’s peak. Thus, the XRD spectrum demonstrates strong evidence in favor of TEM imaging for the presence of Au-NPs.

### EDX analysis

**Fig. 5:**
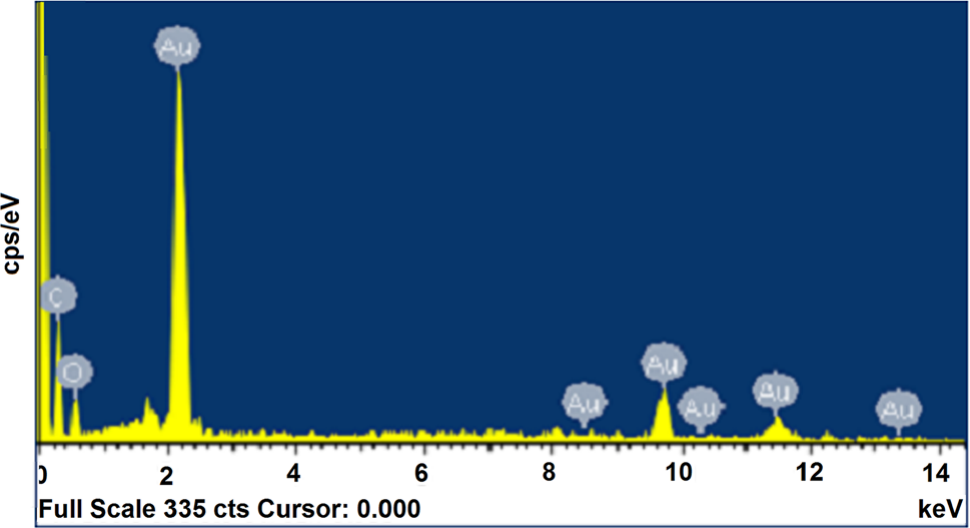
EDX spectrum of biofabricated Au-NPs

EDX analysis was performed to confirm the fabrication of Au-NPs using *D. regia* leaf extract. EDX plot was acquired by plotting kilo electron volt (keV) against count per second/electron volt (cps/eV) (Fig. 5). An intense signal observed at 2.2 keV due to SPR indicated the presence of Au. Elemental signals of C and O were also recorded possibly due to the involvement of carbonyl group of oxidized polyphenols residues in capping and stabilizing of Au-NPs (Hudlikar et al. 2012).

### FTIR analysis

**Fig. 6:**
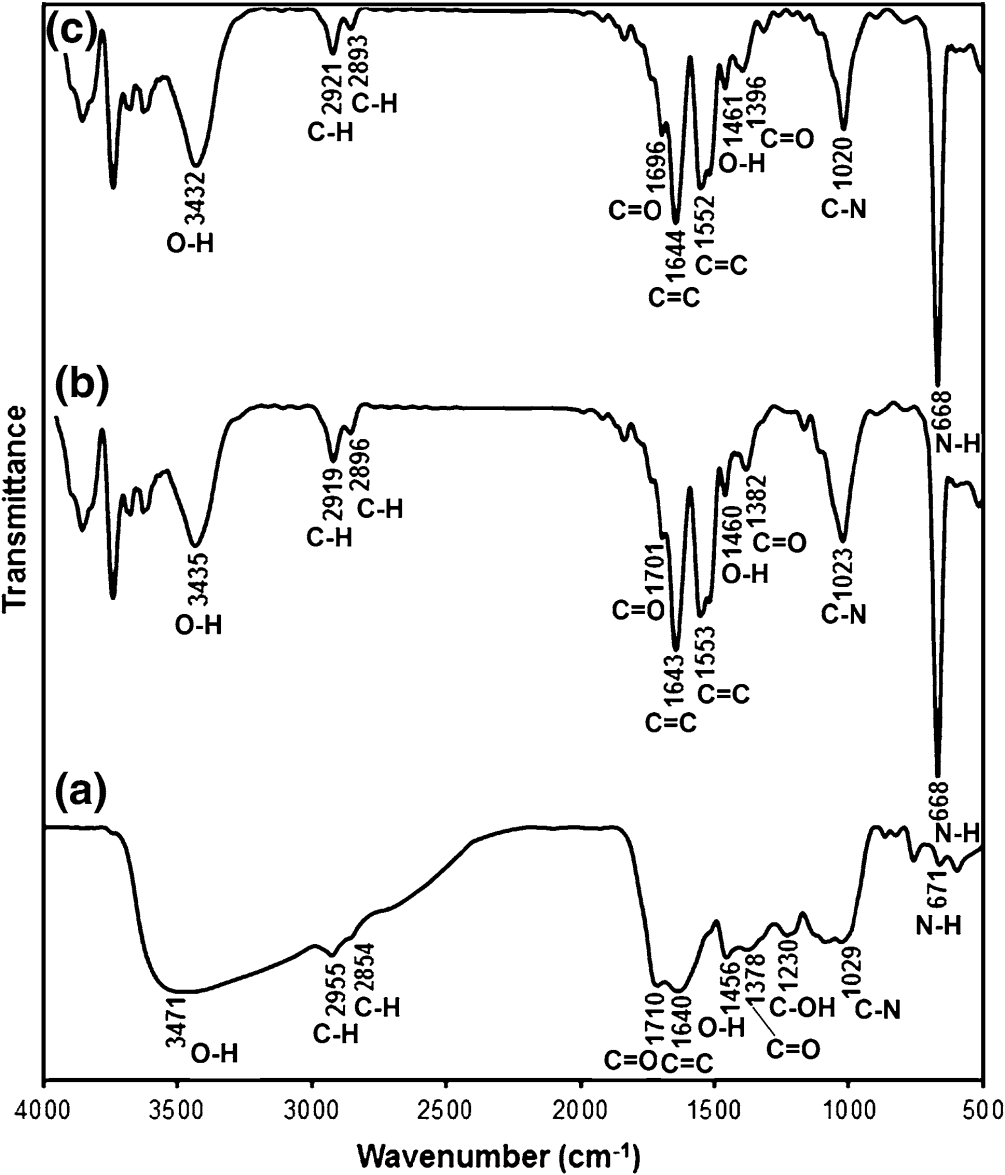
FTIR spectra of (**a**) *D. regia* leaf extract before bioreduction (**b**) *D. regia* leaf extract after bioreduction (**c**) biofabricated Au-NPs

To foresee the involvement of bio-functional groups of leaf extract of *D. regia* in biofabrication of NPs, the FTIR analysis was performed for Au-NPs (Fig. 6). Complex nature of *D. regia* leaf extract (not treated with salt solution) was confirmed from FTIR spectrum of leaf extract, as reported earlier in the literature (Dauthal and Mukhopadhyay 2013b) (Fig. 6a). The FTIR spectra of *D. regia* leaf extract showed absorption bands at 3471, 2925, 1710, 1640, 1456, 1378, and 1230 cm^−1^ represented O–H, C–H, C=O, C=C, O–H, C=O, and C–OH stretching vibration of polyols. These bands indicated that polyols (phenolic acid and flavonoids) are abundant in *D. regia* leaf, as reported earlier (Dauthal and Mukhopadhyay 2013b).

Minor changes were observed in positions and the magnitude of the stretching vibration in the FTIR spectrum of *D. regia* leaf extract after the bioreduction of HAuCl_4_·3H_2_O (Fig. 6b). Significant narrowing observed in the region of 2800–3600 cm^−1^ indicated that the O–H group of polyols participated in the bioreduction reactions. Furthermore, a new stretching vibration was also observed at 1553 cm^−1^ (representing C=C stretching in the aromatic ring). The peaks of 1640, 1456, and 1029 cm^−1^ were shifted to new positions 1643, 1460, and 1023 cm^−1^, respectively, in FTIR spectra of *D. regia* after bioreduction. Changes observed in the FTIR spectrum of *D. regia* leaf extract after bioreduction indicated the participation of polyols having functional groups of O–H, C–H, C=O, C=C, O–H, C=O, and C–OH in bioreduction reactions.

Several peak present at 3432, 2921, 2896, 1696, 1644, 1552, 1461, 1396, 1020, and 668 cm^−1^ positions in FTIR spectrum of Au-NPs also reflected the involvement of different functional groups of phytochemicals of *D. regia* in biofabrication process. The presence of water soluble polyphenolic compounds, including protocatechuic acid, gallic acid and hydroxybenzoic acid (phenolic acid), and catechin (flavonoids), is reported earlier in leaf extract of *D. regia* by the HPLC analysis (Dauthal and Mukhopadhyay 2013b). Metal-reducing potential of these water soluble heterocyclic compounds was also reported earlier (Yoosaf et al. 2007). Functional groups associated with these polyphenolic compounds may thus be involved in reducing the Au^3+^ and stabilizing nanostructures.

**Fig. 7:**
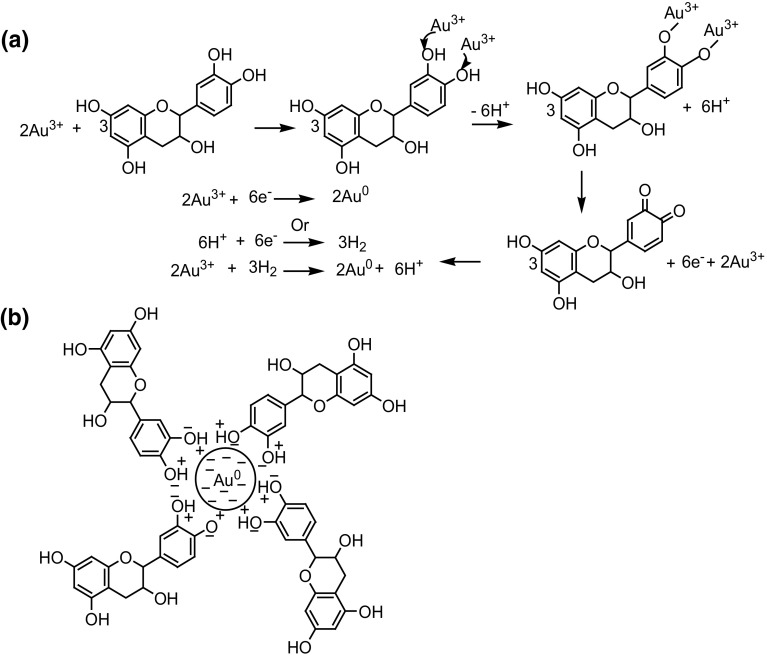
Probable reaction of (**a**) bioreduction and (**b**) stabilization of Au^0^ using representative polyphenol (catechin)

In general, polyphenols present in natural products have at least two hydroxyl groups at para or ortho positions to each other and are reported to have good metal-reducing activity (Yoosaf et al. 2007). All the reported polar polyphenolic compounds of leaf extract possess two hydroxyl groups each. Therefore, all reported polyphenolic compounds may involve in the reduction of Au^3+^ ions and converted into their respective quinones. Oxidized polyphenol (quinones) may act as ligands which facilitates the coordination of carbonyl groups with the freshly fabricated Au-NPs and substantially enhanced their stability (Fig. 7). Similar type of interaction was also demonstrated earlier between carbonyl oxygen atom of Polyvinylpyrrolidone and NPs surface (Zhou et al. 2009).

### Catalytic reduction of *o*-NA

**Fig. 8:**
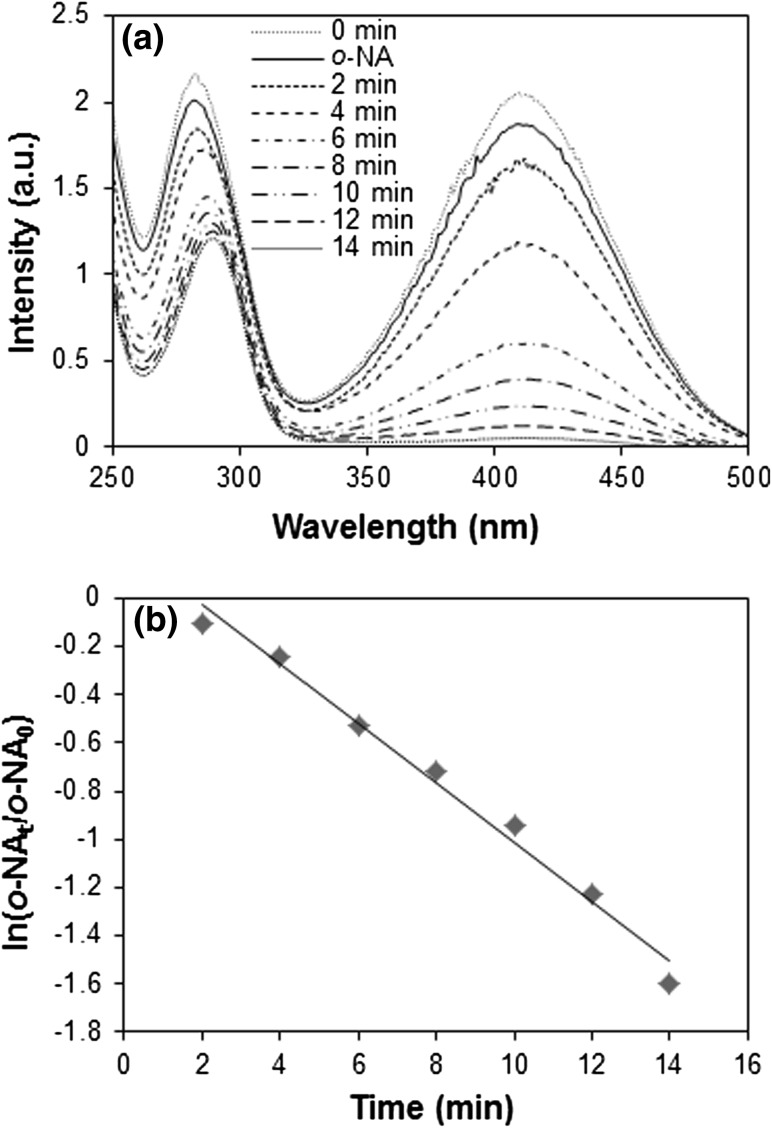
**a** Absorption spectra of catalytic reduction of *o*-NA by NaBH_4_ using biofabricated Au-NPs as catalyst. **b** Pseudo-first-order kinetic plot of In(*o*-NA_t_/*o*-NA_0_) against reaction time *t* (reaction conditions *o*-NA = 0.5 × 10^−3^ M; NaBH_4_ = 1 M; Au-NPs = 1 × 10^−3^ g; temperature 28 ± 2 °C)

Real-time monitoring of catalytic reduction of *o*-NA was done by UV–visible spectroscopy, where characteristic absorbance peak of *o*-NA present at 410 and 283 nm exhibited a progressive decrease, along with slight shift in absorption peak of 283 to 289 nm (Fig. 8a). This reduction reaction of *o*-NA followed three steps, adsorption of *o*-NA onto the surface of catalyst (Au-NPs), interfacial electron transfer, and desorption of the *o*-NA on the surface. Thus, Au-NPs as a catalyst facilitate the reduction reaction of *o*-NA by reducing its activation energy (*E*_a_). A control experiment was carried out devoid of Au-NPs under the same experimental condition. There was no significant change observed in the characteristic *o*-NA peak at 410 nm and did not give any signature of the formation of 1, 2-benzenediamine. However, in the presence of Au-NPs, reduction of *o*-NA followed pseudo-first order, where linear correlation observed between ln(*o*-NA_0_/*o*-NA_*t*_) and reaction time (*t*), where *o*-NA_0_ and *o*-NA_*t*_: absorbance of *o*-NA at the initial time (0) specific time interval (*t*) (Fig. 8b). The pseudo-first-order rate constant (*k*_app_) was calculated from the slope to be 12.28 × 10^−2^ min^−1^.

**Fig. 9:**
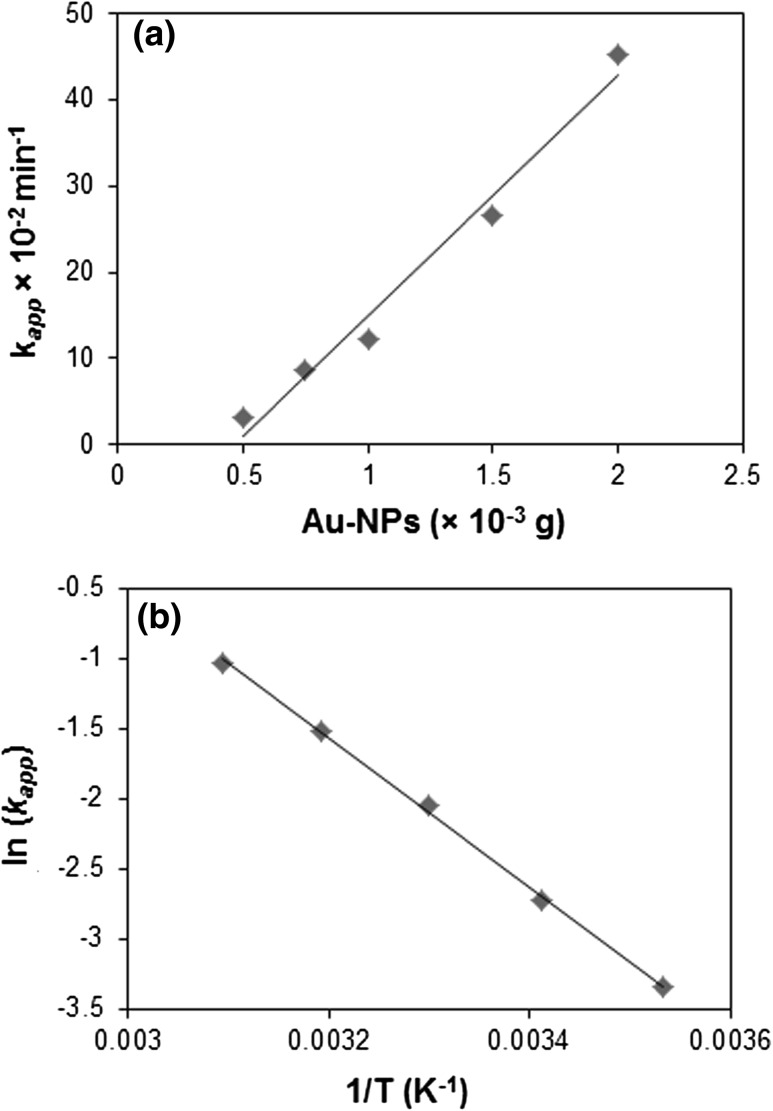
**a** Plot of *k*_app_ against concentration of NPs (reaction conditions: *o*-NA = 0.5 × 10^−3^ M; NaBH_4_ = 1 M; Au-NPs = 0.5–2 × 10^−3^ g; temperature: 28 ± 2 °C). **b** Arrhenius plot for the reduction of *o*-NA over Au-NPs catalyst (reaction conditions *o*-NA = 0.5 × 10^−3^ M; NaBH_4_ = 1 M; Au-NPs = 1 × 10^−3^ g; temperature 10–50 °C)

To study the effect of concentration of catalyst on rate of reduction, the concentration of Au-NPs was varied from 0.5 to 2 × 10^−3^ g, keeping all other parameters constant. Time-dependent reduction of *o*-NA was studied spectrophotometrically by the gradual decrease in the peak intensity at 410 nm. A plot of rate constant (*k*_app_) versus concentration of biofabricated Au-NPs is shown in Fig. 9a. At lowest concentration of Au-NPs (0.5 × 10^−3^ g), the reduction rate of *o*-NA was calculated to be 3.19 × 10^−2^ min^−1^. Reduction rate was increased simultaneously with catalyst concentration, as catalytic reaction usually takes place at the surface of catalyst (Murugadoss and Chattopadhyay 2008). Therefore, the availability of more interaction site at high concentration of NPs ultimately increased the reduction rate.

The effect of temperature in reduction rate of *o*-NA was evaluated in the range of 10–50 °C (Table 1). At 10 °C, *k*_app_ of reduction reaction was found to be 3.52 × 10^−2^ min^−1^, and it was increased to 35.38 × 10^−2^ min^−1^ at 50 °C. The temperature coefficient (*Q*_10_) for this catalytic reduction was found to increase by the factor of 1.6–1.9 for each 10 °C rise in temperature using Eq. 1. Figure 9b represented the Arrhenius plots of *k*_app_ obtained in the range of 10–50 °C. From the slope, the apparent E_*a*_ was calculated to be 44.26 kJ mol^−1^.

**Table 1:** Reduction rate of *o*-NA at different reaction temperatures

*T* (°C)	*K*_app_ × 10^−2^ min^−1^
10	3.52 ± 0.36
20	6.58 ± 0.65
30	12.93 ± 0.94
40	21.73 ± 0.92
50	35.38 ± 1.07

Reaction conditions *o*-NA = 0.5 × 10^−3^ M; NaBH_4_ = 1 M; Au-NPs = 1 × 10^−3^ g

1 $$Q_{10} = \left( {\frac{{k_{\text{app2}} }}{{k_{\text{app1}} }}} \right)^{{{\raise0.7ex\hbox{${10}$} \!\mathord{\left/ {\vphantom {{10} {(T_{2} - T_{1} )}}}\right.\kern-0pt} \!\lower0.7ex\hbox{${(T_{2} - T_{1} )}$}}}}$$

where *k*_app2_ was the apparent rate constant at temperature *T*_2_ and *k*_app1_ was the apparent rate constant at temperature *T*_1_, *T*_1_ and *T*_2_ were the reaction temperatures, and *Q*_10_ represented the factor by which *k*_app_ of a reaction increases for every 10° rise in the temperature.

### Catalytic recyclability of biofabricated Au-NPs

After completion of one cycle of reduction reaction, the used Au-NPs were separated by centrifugation, followed by thorough washing with distilled water and drying. Recovered NPs were again tested for its catalytic activity for the same reaction. To demonstrate easy recovery of the NPs from the reaction, volume of reagents and catalysts was increased by ten times. Slight change observed in *k*_app_ of the recycled catalysts up to five cycles (Table 2). *k*_app_ obtained at the fifth cycle was found to be 11.39 × 10^−2^ min^−1^, suggested catalytic ability of *D. regia*-mediated Au-NPs remained at about 92 % even after recycling for five consecutive cycles. The slight decrease in catalytic activity of NPs suggested agglomeration of NPs (Kuroda et al. 2009; Koga and Kitaoka 2011) or not fully recovered during recycling process. Thus, biofabricated Au-NPs proved as a potent recyclable catalyst for industrial applications.

**Table 2:** Recyclability of biofabricated Au-NPs as catalyst for five cycles

Cycle	*k*_app_ × 10^−2^ (min^−1^)
1	12.28
2	12.19
3	11.94
4	11.56
5	11.39

Reaction conditions *o*-NA = 0.5 × 10^−3^ M; NaBH_4_ = 1 M; Au-NPs = 1 × 10^−3^ g; temperature 28 ± 2 °C

## Conclusions

Phyto-synthesis of Au-NPs has been carried out using metal-reducing potential of aqueous *D. regia* leaf extract. Process variables, such as leaf extract concentration, incubation temperature, time, and pH, are important key factors in controlling the SPR, size, and stability of NPs. Spectroscopic (UV–visible and FTIR), structural (XRD), and morphological (TEM) analysis suggest the key role of polyphenolic compounds of *D.* *regia* in the rapid fabrication of crystalline, pure phase, and spherical Au-NPs of 4–24 nm size through the electrostatic interaction of hydroxyl and carbonyl groups. UV–visible spectroscopy results suggested that biofabricated Au-NPs are a promising candidate for catalytic applications. Therefore, the described strategy for the fabrication of Au-NPs is straightforward, eco-friendly, cost-efficient, and shows a significant great catalytic potential.

## References

[CR1] Agnihotri M, Joshi S, Kumar AR, Zinjarde S, Kulkarni S (2009). Biosynthesis of gold nanoparticles by the tropical marine yeast *Yarrowia lipolytica* NCIM 3589. Mater Lett.

[CR2] Ahmad N, Sharma S, Alam MK, Singh VN, Shamsi SF, Mehta BR, Fatma A (2010). Rapid synthesis of silver nanoparticles using dried medicinal plant of basil. Colloids Surf B.

[CR3] Alani F, Moo-Young M, Anderson W (2012). Biosynthesis of silver nanoparticles by a new strain of *Streptomyces* sp. compared with *Aspergillus**fumigatus*. World J Microbiol Biotechnol.

[CR4] Bai X, Gao Y, Liu HG, Zheng L (2009). Synthesis of amphiphilic ionic liquids terminated gold nanorods and their superior catalytic activity for the reduction of nitro compounds. J Phys Chem C.

[CR5] Dauthal P, Mukhopadhyay M (2012). *Prunus domestica* fruit extract-mediated synthesis of gold nanoparticles and its catalytic activity for 4-nitrophenol reduction. Ind Eng Chem Res.

[CR6] Dauthal P, Mukhopadhyay M (2013). In-vitro free radical scavenging activity of biosynthesized gold and silver nanoparticles using *Prunus armeniaca* (apricot) fruit extract. J Nanopart Res.

[CR7] Dauthal P, Mukhopadhyay M (2013). Biosynthesis of palladium nanoparticles using *Delonix regia* leaf extract and its catalytic activity for nitro-aromatics hydrogenation. Ind Eng Chem Res.

[CR8] Dauthal P, Mukhopadhyay M (2015). Biofabrication, characterization, and possible bio-reduction mechanism of platinum nanoparticles mediated by agro-industrial waste and their catalytic activity. J Ind Eng Chem.

[CR9] Dauthal P, Mukhopadhyay M (2015). Agro-industrial waste-mediated synthesis and characterization of gold and silver nanoparticles and their catalytic activity for 4-nitroaniline hydrogenation. Korean J Chem Eng.

[CR10] Dauthal P, Mukhopadhyay M (2016). AuPd bimetallic nanoparticles: single step biofabrication, structural characterization and catalytic activity. J Ind Eng Chem.

[CR11] Gangula A, Podila R, Ramakrishna M, Karanam L, Janardhana C, Rao AM (2011). Catalytic reduction of 4-nitrophenol using biogenic gold and silver nanoparticles derived from *Breynia rhamnoides*. Langmuir.

[CR12] Gardea-Torresdey JL, Gomez E, Peralta-Videa JR, Parsons JG, Troiani H, Jose-Yacaman M (2003). Alfalfa sprouts: a natural source for the synthesis of silver nanoparticles. Langmuir.

[CR13] Hudlikar M, Joglekar S, Dhaygude M, Kodam K (2012). Green synthesis of TiO_2_ nanoparticles by using aqueous extract of *Jatropha curcas* L. latex. Mater Lett.

[CR14] Jacob SJP, Finub JS, Narayanan A (2012). Synthesis of silver nanoparticles using *Piper longum* leaf extracts and its cytotoxic activity against Hep-2 cell line. Colloids Surf B.

[CR15] Koga H, Kitaoka T (2011). One-step synthesis of gold nanocatalysts on a microstructured paper matrix for the reduction of 4-nitrophenol. Chem Eng J.

[CR16] Kuroda K, Ishida T, Haruta M (2009). Reduction of 4-nitrophenol to 4-aminophenol over Au nanoparticles deposited on PMMA. J Mol Catal A: Chem.

[CR17] Liu W, Yang X, Xie L (2007). Size-controlled gold nanocolloids on polymer microsphere-stabilizer via interaction between functional groups and gold nanocolloids. J Colloid Interface Sci.

[CR18] Metuku RP, Pabba S, Burra S, Bindu NSVSSSLH, Gudikandula K, Charya MAS (2014). Biosynthesis of silver nanoparticles from *Schizophyllum radiatum* HE 863742.1 their characterization and antimicrobial activity. 3 Biotech.

[CR19] Murugadoss A, Chattopadhyay A (2008). Surface area controlled differential catalytic activities of one-dimensional chain-like arrays of gold nanoparticles. J Phys Chem C.

[CR20] Rajathi FAA, Parthiban C, Kumar VG, Anantharaman P (2012). Biosynthesis of antibacterial gold nanoparticles using brown alga, *Stoechospermum marginatum* (kützing). Spectrochim Acta A Mol Biomol Spectrosc.

[CR21] Sathiya CK, Akilandeswari S (2014). Fabrication and characterization of silver nanoparticles using *Delonix elata* leaf broth. Spectrochim Acta A Mol Biomol Spectrosc.

[CR22] Sharma NC, Sahi SV, Nath S, Parsons JG, Gardea-Torresde JL, Pal T (2007). Synthesis of plant-mediated gold nanoparticles and catalytic role of biomatrix-embedded nanomaterials. Environ Sci Technol.

[CR23] Singh P, Kim YJ, Wang C, Mathiyalagan R, Yang DC (2015). The development of a green approach for the biosynthesis of silver and gold nanoparticles by using *Panax ginseng* root extract, and their biological applications biological applications. Artif Cells Nanomed Biotechnol.

[CR24] Slocik JM, Naik RR, Stone MO, Wright DW (2005). Viral templates for gold nanoparticle synthesis. J Mater Chem.

[CR25] Srivastava N, Mukhopadhyay M (2014). Biosynthesis of SnO_2_ nanoparticles using bacterium *Erwinia herbicola* and their photocatalytic activity for degradation of dyes. Ind Eng Chem Res.

[CR26] Suresh AK, Pelletier DA, Wang W, Broich ML, Moon JW, Gu B, Allison DP, Joy DC, Phelps TJ, Doktycz MJ (2011). Biofabrication of discrete spherical gold nanoparticles using the metal-reducing bacterium *Shewanella oneidensis*. Acta Biomater.

[CR27] Tripathy A, Raichur AM, Chandrasekaran N, Prathna TC, Mukherjee A (2010). Process variables in biomimetic synthesis of silver nanoparticles by aqueous extract of *Azadirachta indica* (Neem) leaves. J Nanopart Res.

[CR28] Wang C, Kim YJ, Singh P, Mathiyalagan R, Jin Y, Yang DC (2015). Green synthesis of silver nanoparticles by *Bacillus methylotrophicus*, and their antimicrobial activity. Artif Cells Nanomed Biotechnol.

[CR29] Yoosaf K, Ipe BI, Suresh CH, Thomas KG (2007). In situ synthesis of metal nanoparticles and selective naked-eye detection of lead ions from aqueous media. J Phys Chem C.

[CR30] Zayed MF, Eisa WH (2014). *Phoenix dactylifera* L. leaf extract phytosynthesized gold nanoparticles; controlled synthesis and catalytic activity. Spectrochim Acta A Mol Biomol Spectrosc.

[CR31] Zayed MF, Eisa WH, Abdel-Moneam YK, El-kousy SM, Atia A (2015). *Ziziphus spina*-*christi* based bio-synthesis of Ag nanoparticles. J Ind Eng Chem.

[CR32] Zhou M, Wang B, Rozynek Z, Xie Z, Fossum JO, Yu X, Raaen S (2009). Minute synthesis of extremely stable gold nanoparticles. Nanotechnology.

